# Lipid-Based Nanocarrier System for the Effective Delivery of Nutraceuticals

**DOI:** 10.3390/molecules26185510

**Published:** 2021-09-10

**Authors:** Parthasarathi Subramanian

**Affiliations:** Riddet Institute, Massey University, Private Bag 11222, Palmerston North 4442, New Zealand; p.subramanian@massey.ac.nz

**Keywords:** bioavailability, liposomes, niosomes, physicochemical stability, solid lipid nanoparticles, polyphenols

## Abstract

Nutraceuticals possess several health benefits and functions; however, most nutraceuticals are prone to degradation in the gastrointestinal environment and have poor bioavailability. Application of a novel carrier system is of increasing importance to overcome obstacles and provide efficient applicability. Lipid-based nanocarriers provide a large surface-to-mass ratio, enhanced intestinal absorption by solubilization in the intestinal milieu, intestinal lymphatic transport, and altering enterocyte-based transport. A critical overview of the current limitation, preparation, and application of lipid-based nanocarriers (liposomes and niosomes) and lipid nanoparticles (SLNs and NLCs) is discussed. Physical and gastrointestinal stability and bioavailability of nanoencapsulated nutraceuticals are considered as well.

## 1. Introduction

The interest of public and health professionals has been drawn to the importance of nutraceuticals for the prevention of chronic diseases and has resulted in a huge demand for nutraceutical products. The translation of nutraceutical compounds from lab to market is very tedious, which involves a long and extensive journey through basic research, formulation, complicated clinical trials, and regulatory approvals. According to the Global Opportunity Analysis and Industry Forecast, 2014–2022, the demand for functional foods (including nutraceuticals) and beverages has significantly increased in the past few years due to the increase in the cost of synthetic pharmaceutical active compounds. Further, the global nutraceutical market is expected to reach USD 302,306 million by 2022 from USD 184,092 million in 2015 with a CAGR of 7.04% from 2016 to 2022. The global nutraceutical product market is profitable due to the increase in awareness among consumers of certain nutraceuticals, such as omega-3-fatty-acid-fortified food and herbal extracts.

Oral delivery is the most convenient and widely acceptable route of administration due to ease of administration and cost-effectiveness in the formulation. The success of nutraceutical compounds is owing to numerous factors, such as therapeutic effect, physicochemical stability, and bioavailability. However, many bioactive compounds suffer from poor bioavailability due to poor solubility in the gastrointestinal tract, limited intestinal permeability, and hepatic first-pass metabolism. To reach the bloodstream, the bioactive compound should first dissolve in the gastrointestinal fluid and maintain its stability, but poorly water-soluble compounds (lipophilic bioactive compounds) result in poor absorption and low bioavailability due to the poor dissolution characteristics. Metabolism (intestinal and first-pass metabolism) is the second factor that restricts bioavailability to a significant extent [1]. Several approaches such as coacervation, liposome entrapment, inclusion complexation, cocrystallization, and emulsification have been evaluated for the improvement of the bioaccessibility and bioavailability of nutraceutical compounds. These technological and chemical modifications of the nutraceutical molecules are aimed at improving their solubility, the site of absorption, the design of colloidal systems (micelles and vesicles), and the use of nanosystems [2]. A nanotechnology (nanocarrier)-based delivery system is one of the major formulation research studies providing direct benefit to human health through clinical and commercial development [3]. These nanocarriers may lead to better half-life, precise release-completed short or extended durations, and identical site-specific targeted delivery of therapeutic compounds [4,5]. Different materials have been used for the construction of nanocarriers, including polymers, metals, and lipids. Among these, a lipid-based carrier is the most suitable technique in the food and nutraceutical sector because of its biocompatibility and biodegradability [6]. Although lipid nanocarriers cover a vast technique, only selected lipid nanocarriers were reported for its successful commercialization, including nanoemulsions, liposomes, solid lipid nanoparticles (SLNs), nanostructured lipid carriers (NLCs), niosomes, and self-emulsifying drug delivery systems (SEDDSs) [7]. A short summary of the selected lipid-based delivery system is presented in Table 1.

A wide range of essential bioactives/nutraceuticals in nature possess hydrophobic characteristics, including phenolic compounds, essential oils and fatty acids, carotenoids, and insoluble vitamins, and such hydrophobic bioactives are essential for the human health. These poorly soluble characteristics of bioactive compounds eventually reduce the bioavailability and sustainability [8]. A lipid-based carrier can be a favorable approach for protecting bioactive compounds in the gastrointestinal (GI) tract and enhancing their bioavailability. Thus, the object of this manuscript is to highlight the potential of lipid-based nanocarriers for the effective delivery of nutraceuticals. However, these lipid-based nanocarriers cover a wide range of techniques, including liposomes, ethosomes, Pickering emulsions, nanoemulsions, and solid lipid nanoparticles. This review was limited to the preparation methods, stability, and absorption mechanism of vesicular nanocarriers (liposomes and niosomes) and particulate systems (SLNs/NLCs).

## 2. Overview of Digestion

Food digestion is a complex process that involves the transit of food through the mouth, stomach, and small intestine. During digestion, the ingested food is broken down mainly by two processes: (i) mechanical breakdown, where solid foods of larger particles are broken down into smaller pieces, and (ii) enzymatic breakdown, where several digestive enzymes act on the food bolus during the gastrointestinal transit. The secretion of digestive enzymes initiates in the oral phase and continues in the stomach and intestine [9]. Solid and semisolid foods require considerable physical transformation during digestion.

The oral cavity is the first site of food digestion, involving mechanical grinding (physical disruption by chewing) and enzymatic hydrolysis by mixing with saliva. Salivary glands in the oral cavity secret saliva, which is near neutral pH with salivary amylase enzyme to hydrolyze starch molecules into glucose. During oral digestion, the food is broken down into small pieces, is mixed with saliva, and forms oral bolus [10].

Gastric digestion: Oral bolus masticated with amylase is then transported to the gastric compartment through the esophagus by peristalsis motion. The stomach accommodates the bolus within four regions—cardiac, fundic, body, and pylorus—and converts masticated bolus into semisolid chyme [11]. Briefly, the proximal stomach (upper part of the stomach) acts as a receiving center/reservoir for the oral bolus/undigested food. Additionally, the distal part is responsible for the breaking of solid foods by grinding, propelling, and mixing the undigested food with gastric secretions for enzymatic breakdown. In a recent study, Sutter, et al. [12] reported that the rate of gastric contraction ranges between 2.6 and 3.8 times per minute, and the median contraction frequency is higher in women (3.2 times per minute) and lower in men (3.0 times per minute). Further, gastric secretion consists of hydrochloric acids, electrolyte salts, mucus, enzymes such as gastric lipase (digest fats) and pepsin (to digest protein), and various hormones. The pH of the stomach dynamically changes between 1.5 and 2.0 in the fasted state and 3.0 and 7.0 in the fed state [13]. The digested food leaves the stomach through the pylorus, which acts as a sieve and allows food with 1–2 mm size, and this process is called gastric emptying. Additionally, the food material that does not achieve <2 mm size is maintained in the distal part by the retropulsion mechanism and undergoes the grinding process until it achieves the desired size range.

Small intestine: Disintegrated food with <2 mm size will move to the small intestine through the pylorus for digestion and nutrient absorption. The small intestine comprises three compartments: duodenum, jejunum, and ileum. The duodenum possesses a short length (~30 cm) and is located immediately next to the stomach, where the digesta from acidic pH moves to neutral pH. In this region, the hydrolytic enzyme pancreatin secreted by the pancreas mixes with the digesta and further hydrolyzes the proteins, fats, and carbohydrates. Further, the bile acids introduced through the biliary tract into the small intestine emulsify the fat droplets and help in the digestion and absorption of fats and fat-soluble vitamins. Intestinal motility, including peristalsis and segmentation, plays a predominant role in mixing and transporting the digesta across the intestine [14].

In the case of lipid-based nanocarriers, it provides distinctive features, including large surface-to-mass ratio, long-term stability, modification and conjugation capability, and encapsulation of nutraceutical molecules [15]. Lipid-based formulations can significantly enhance the intestinal absorption of poorly water-soluble compounds. Porter, et al. [16] reported three main mechanisms by which lipophilic excipients enhance the absorption:

1. Enhancing bioactive solubilization in the intestinal milieu: Dissolution is a prerequisite for the absorption of nutraceuticals from the small intestinal lumen into the enterocytes (intestinal absorptive cells). Lipid-based nanocarriers enhance the solubilization in the GI tract by providing the bioactive compounds in a solubilized form and preventing precipitation in the intestinal milieu.
2. Intestinal lymphatic transport: Highly lipophilic molecules can be transported to the systemic circulation through the intestinal lymphatic system. In normal cases, the portal vein is employed for the transport of molecules (through first-pass metabolism) to the systemic circulation. Lipid-based systems recruit endogenous and exogenous lipid transport and stimulate intestinal lymphatic transport of coadministered lipophilic bioactive compounds, thereby protecting the molecules from the first-pass metabolism.
3. Altering enterocyte-based bioactive transport: Lipids and lipophilic excipients can alter the expression of intracellular lipid-binding proteins by interacting with apical membrane lipid transporters and modify the intracellular pooling of lipids within the enterocyte.

**Figure 1:**
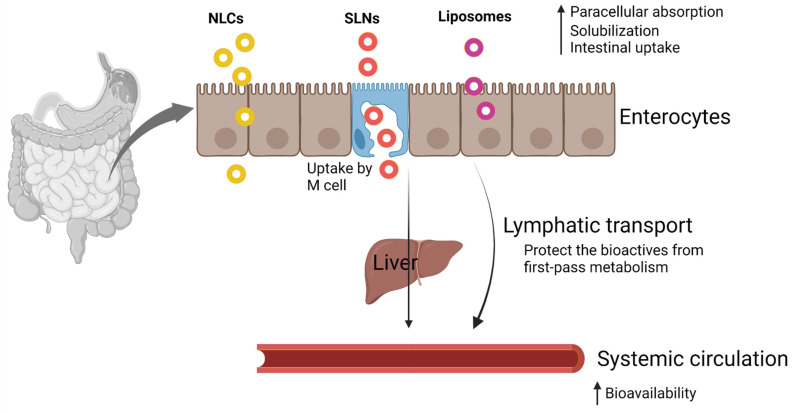
Transport mechanism of nanosized lipid-based delivery systems (created with Biorender.com).

Various transfer mechanisms, such as paracellular absorption, M-cell-mediated transport, and endocytosis, are involved in the transportation of nanosized lipids across the intestinal membrane (Figure 1).

Paracellular absorption: Orally administered nutraceuticals must reach the systemic circulation via absorption through the intestinal epithelial layer. There are four different transfer mechanisms: paracellular, transcellular, carrier-mediated, and receptor-mediated transport. Paracellular absorption refers to the transport of molecules between adjacent epithelial cells, but the rate-limiting step in this absorption process is the transport across the tight junction [17]. The intestinal epithelium is one of the major barriers to the absorption of hydrophilic bioactive molecules due to the nondiffusional properties of epithelial cells through lipid bilayer cell membranes to the bloodstream [18]. Ji, et al. [19] enhanced the solubility and bioavailability of curcumin by solid lipid nanoparticle formulation with TPGS and Brij78 (P-gp modulator). Intestinal effective permeability was significantly improved for curcumin SLNs, thereby increasing the relative bioavailability to 942.53% compared with the curcumin suspension.

M-cell-mediated transport: M-cells (also called as macrofold cells) are specialized intestinal epithelial cells found in the follicle-associated epithelium of intestinal Peyer’s patches of gut-associated lymphoid tissue and isolated lymphoid follicles responsible for the immune sensing of luminal bacteria [20,21]. The role of M-cells includes nutrient absorption from the intestine and delivery of intestinal microbial antigens to gut-associated lymphoid tissue for mucosal and systemic immune responses [21].

Endocytosis: All intestinal cells use endocytosis to take up nutrients from the external milieu by four separate mechanisms: clathrin-mediated endocytosis, caveolae, micropinocytosis, and phagocytosis. Clathrin- and caveolin-mediated endocytoses involve the receptor binding of macromolecules [22]. In the clathrin-mediated endocytosis, nanoparticles interact with receptors on the cytomembrane (clathrin-1) and form clathrin-coated vesicles (CCV) through the GTPase activity of dynamin. CCVs penetrate inside the cells through the energy supplied by actin, and the destination of the CCVs is decided by the receptor that is attached to the ligands of nanoparticles [23]. Macropinocytosis is a form of endocytosis that accompanies cell surface ruffling and is mediated by the actin cytoskeleton that internalizes the surrounding fluid into large vacuoles [24,25]. The diameter of the endocytotic vesicles can be larger than those of clathrin- and caveolin-mediated endocytoses. Chai, et al. [26] first revealed the transport mechanisms of solid lipid nanoparticles (SLNs) across intestinal epithelial cell monolayers (Caco-2 cells). Internalization of SLNs was mediated by the macropinocytosis pathway and clathrin- and caveolae (or lipid raft)-related routes. Further, transcytosis (transcellular transport) of SLNs did not damage the tight junctions of intestinal epithelial cells and ensures SLNs as a safe delivery system for oral administration of bioactive compounds.

**Figure 2:**
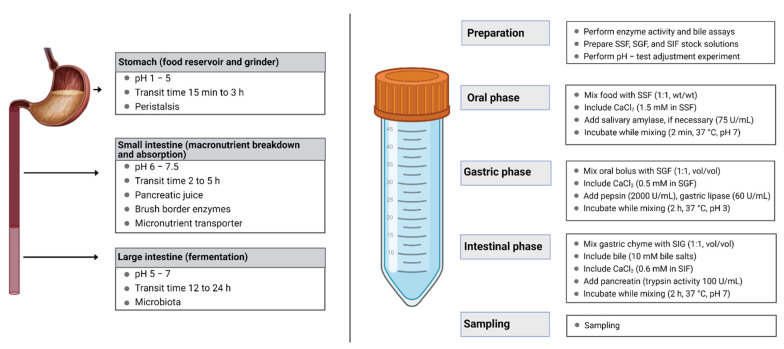
Region specificity and role of gastrointestinal tract during food digestion (**left**) and flow diagram of in vitro INFOGEST digestion protocol (**right**). This figure is reproduced from Li, et al. [31].

Need for digestion studies: In recent years, there is an increasing awareness among consumers of the food they are consuming, including innovative functional food products and nutraceutical formulations. A thorough understanding of the digestion process from the particle breakdown at the oral phase to the nutrient absorption at the intestinal phase is necessary to design effective food products [27]. Figure 2 illustrates an overview of in vivo and in vitro digestion models.

The evolution of food-grade delivery systems is showing promising results in improving the stability, bioavailability, and release characteristics of nutrients. These delivery systems are designed in such a way that the encapsulated nutrient should be released/exposed at the specific location in the GI tract, often by trigger factors, such as ionic strength, pH, or enzyme activity [28]. Testing the efficiency of such formulations is much necessary before introducing those products to the market. Digestion and absorption studies involving human volunteers (clinical trials) are still considered the ‘golden standard’ for addressing the response of a specific diet/formulation in the human gastrointestinal system. However, such in vivo studies are complex, labor-intensive, and expensive and undergo severe ethical restrictions [29]. In recent years, a considerable amount of effort has been devoted to developing in vitro models that provide an easy sampling technique and rapid digestion and simulate physiological factors such as pH, salt concentration, and digestive enzymes. Dynamic in vitro models can simulate experimental conditions similar to in vivo physiological conditions and widely accepted in food and pharmaceutical industries for their accuracy and reproducibility of data [30].

## 3. Vesicular Nanocarriers

**Figure 3:**
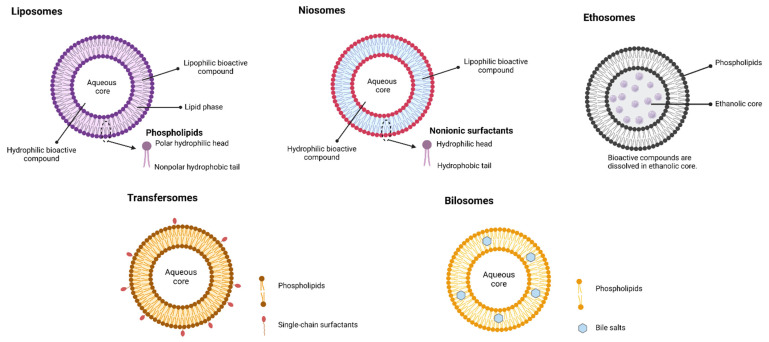
Classification of vesicular systems (created with Biorendor.com).

Vesicular nanocarriers are colloidal carrier systems showing great promise in drug/nutraceutical delivery. Vesicular nanocarriers have been extensively investigated in the past few decades for pharmaceutical applications and revealed improved pharmacokinetics, solubility, stability, and biodistribution; decreased toxicities; controlled release; and site-specific delivery of therapeutic agents [32,33]. Vesicular systems are useful to encapsulate both hydrophilic (interior hydrophilic compartment) and lipophilic (in outer lipid layer) bioactive compounds. Vesicular nanocarriers are classified based on their principal compounds (see Figure 3). In this manuscript, only the application of liposomes and niosomes was reviewed due to their promising application in the food sector.

### 3.1. Liposomes

Liposomes are phospholipid bilayered vesicular systems having an aqueous core enclosed by one (unilamellar) or several (multilamellar) concentric phospholipid membranes [34,35]. Mozafari, et al. [36] proposed a definition for liposomes as ‘closed, continuous bilayered structures made mainly of lipid and/or phospholipid molecules’. To avoid confusion among other lipid-based delivery systems with liposomes, the US Food and Drug Administration (USFDA) clearly defined that liposomes are not emulsion systems or a drug–lipid complex [37].

**Figure 4:**
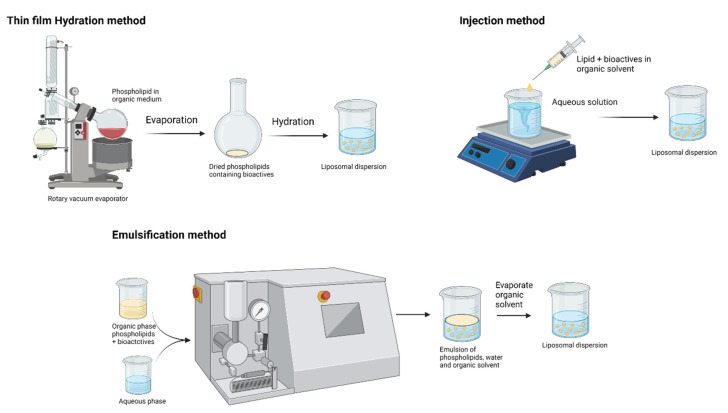
Production techniques of liposomes (created with Biorendor.com).

Liposomes vary with composition, size, surface charge, and method of preparation. The main attractive feature in the application of liposomes is that they can accommodate both water-soluble (in the aqueous core region) and lipid-soluble (in lamellae) bioactive compounds [32]. Schematic representations of liposome preparation techniques are shown in Figure 4. These biodegradable and biocompatible compositions of liposomes make them excellent carriers of therapeutic agents. Conventional liposome preparation involves the following stages: drying of lipid/phospholipid ingredients from organic solvents, dispersion in aqueous media (it creates vesicles), purification of the resultant vesicles, and analysis of the final product. Thus, the increasing interest in lipid-based nutraceutical formulation can be correlated with the increasing number of clinical trials. Table 2 summarizes the recent clinical trials on lipid-based formulations. However, the traces of organic solvent residues may remain in the lipid and/or aqueous phase and result in toxicity. Advances in the field of encapsulation technology have brought a possible way to prepare lipid vesicles without volatile organic solvents. It is a remarkable development by Prof. Mozafari to formulate microcapsules without the application of toxic solvents, detergents, and harsh treatments such as sonication/microfluidization [38,39]:

1. Add nanoliposomal ingredients to the mixture (preheated to 40–60 °C) of active agent and polyol in a heat-resistant vessel.
2. Heat the mixture at 40–60 °C while stirring in an inert atmosphere (pass nitrogen or argon gas to create an inert atmosphere).
3. Keep the product (nanoliposome) above the phase transition temperature of the phospholipid ingredients under an inert atmosphere for 1 h so that the nanoliposomes will anneal and stabilize. Then bring the nanoliposome to an ambient temperature gradually and store.

#### 3.1.1. Physicochemical Stability

The increasing interest in the liposomal delivery system is due to its functional properties, including efficient encapsulation capacity and biocompatibility with food constituents. However, the main problem associated with the industrial application of liposomes is their insufficient physiochemical stability due to the fragile bilayer phospholipid membranes and oxidation or hydrolysis of the fatty acids. One strategy to improve the physiochemical stability is the application of polysaccharides in liposomal preparation. The addition of polysaccharides (modifying surface charges) and antioxidants to liposomes minimizes the oxidative degradation of liposomes, including high-quality lecithins with low levels of hydroperoxides and transition metals [43]. Lopes, et al. [44] investigated the physicochemical properties and stability of the nisin (antibacterial agent) liposomes covered with polysaccharides (polygalacturonic acid/pectin). Polygalacturonic acid liposomes were stable for over 28 days, with a polydispersity index (PDI) of around 0.2 to 0.3, which indicates that the negatively charged polysaccharides increased the electrostatic repulsion between nanoparticles and thereby increased their stability. Later, the same group coencapsulated nisin and lysozyme in polysaccharide-coated liposomes (polygalacturonic acid/pectin). Polysaccharide-coated liposomes maintained constant particle and zeta potential due to the presence of polysaccharide layers on the surface or induced stabilization through the reduction of charges on the polysaccharide and liposome due to electrostatic screening [45].

Azzi, et al. [46] reported the higher encapsulation capacity and improved physicochemical properties of quercetin encapsulated with cyclodextrin-coated liposomes. Quercetin is a flavonoid known for its beneficial effects, including antiobesity, anticancer, anti-inflammatory, and antioxidant properties. However, poor water solubility and poor chemical stability are the main drawbacks of its application in the food sector. Due to poor aqueous solubility, quercetin possesses low bioavailability and a short biological half-life [47]. The authors revealed that the quercetin-loaded liposomes were stable (evaluated through particle size and quercetin content) even after 12 months of storage (@ 4 °C). Surprisingly, a higher amount of quercetin (98%) was retained after 12 months in the quercetin in cyclodextrin in liposomes. Pu, et al. [48] came up with a different strategy with cationic guar gum (CGG) to increase the stability of curcumin. CGG is the cationic quaternary group of guar gum (GG) and has a good affinity for negatively charged compounds. As a coating material, nonionic GG might coat the negatively charged liposomes with physical absorption, but the cationic CGG is absorbed on the negative surface of the liposomes via electrostatic interaction and remains stable. The negative charge of the liposomal surface was neutralized by the CGG interactions and turned the net charge positive. The authors observed the better protection effect (able to provide higher curcumin retention at 70 °C) of curcumin-loaded liposomes coated with CGG. On the other hand, nanoliposomes have been reported as a promising delivery system for transporting food bioactive compounds.

Plant-based phospholipids (PLs), such as soybean, rapeseed, sunflower PLs, were frequently used for liposomal preparation, and the major PL components include zwitterionic phosphatidylcholine (PC), phosphatidylethanolamine (PE), and anionic phosphatidylinositol (PI). However, those plant-based PLs are more highly susceptible to oxidation and lower physical and storage stability owing to the high degree of unsaturation [49]. Alternatively, animal-based PLs, such as eggs, milk, meat, and marine source, are gaining attention for the fabrication of liposomes. Interestingly, marine PLs (fish roe, krill oil, and fish) are reported for higher PUFAs, which makes them a promising functional ingredient [50]. Milk PLs contain high levels of sphingomyelin (SM) in addition to other PL components and are reported to have a higher degree of saturation. Wu, Mou, Song, Tan, Lai, Shen and Cheong [49] studied the effect of bovine milk and krill PLs for curcumin-loaded liposomes and noticed that the liposome of bovine milk PLs exhibited higher stability with a smaller particle size and greater zeta potential (163 nm and −26.7 mV) than the krill PL liposomes (212 nm and −15.23 mV). Both curcumin liposomes were stable (curcumin retention: 80%) after 40 h exposure in acidic conditions, but the retention rate of curcumin was reduced to 50% at neutral and alkaline conditions. Additionally, curcumin retention is higher in bovine milk PL formulation than the krill PLs attributed to the high amount of phosphatidylcholine in krill PLs, which are susceptible to hydrolysis, resulting in the leakage of curcumin from the phospholipids.

#### 3.1.2. Enhancing the Bioavailability of Polyphenols by Liposomal Technology

There is always a demand for plant-derived bioactive compounds, which are considered natural and safer. Polyphenols are the secondary plant metabolites, which include nearly 10,000 different compounds with one or more aromatic rings and hydroxyl groups. Readers can refer to Brglez Mojzer, et al. [51] for an understanding of the polyphenols and the different extraction methods. Polyphenols are generally classified as flavonoids and phenolic acids. Additionally, flavonoids are further divided into flavones, flavonols, flavanones, and isoflavones, and phenolic acids as hydroxybenzoic and hydroxycinnamic acids [52]. However, the poor absorption and low bioavailability of extracted polyphenols restrict the wide application of polyphenol-based functional foods. Entrapping those polyphenols in the bilayered vesicular systems (liposomes) is one of the ideal ways to improve GI stability and bioavailability.

However, conventional liposomes or nanoparticles do not cross the intestinal mucosal barrier due to their relatively large size [53], are unstable in the gastric environment, and release the entrapped bioactive compounds prior to the target site. From the previous research, it is evident that the bioactive compounds need to be absorbed/transformed into mixed micelles in the GI tract and transported to the epithelial cells. M-cell-mediated transport is reported for the possible absorption route for liposomes and nanoparticles; however, the M-cells may account for 1% of the total epithelial cell population [54]. The following strategies were employed for enhancing the intestinal absorption of encapsulated bioactive compounds.

Curcumin: Curcumin is a polyphenolic compound reported for its antioxidant, anti-inflammatory, antimicrobial, and anticancer properties. However, the wide application of curcumin is limited because of its poor aqueous solubility, rapid metabolism, and low permeability due to its susceptibility to P-glycoprotein efflux [55]. The stability of curcumin in the liposomes can be improved further by converting the liquid formulation into a dry form. Additionally, conventionally either spray drying or freeze drying is used for this purpose. Alternatively, Gopi, et al. [56] prepared liposomal curcuminoids in powder form through a novel nanofiber weaving technology. The release of curcumin from the liposomal curcuminoid powder was evaluated at pH 5.5 (46.2%) and pH 7.4 (40.4%), and the burst release (initial rupture) was observed within 12 h and then followed a slow and sustained release. A slightly acidic pH accelerated the release of curcumin from the formulation, and the strong interaction between phospholipids and curcumin at pH 7.4 showed less desorption.

Recent research on Pluronic-modified liposomes showed promising results in maintaining the stability of bioactives in the GI tract and improving bioaccessibility. Pluronics (also called poloxamer, Kolliphor, and Synperonic) are nonionic triblock copolymers with a central hydrophobic poly (propylene oxide (PPO)) chain with two hydrophilic poly (ethylene oxide (PEO)) on each side [57]. Pluronic-modified liposomes protected the curcumin from degradation, and nearly 50% of curcumin was retained after exposure to 80 °C. Additionally, the bioaccessibility of curcumin was increased to 43.3%, whereas the simple curcumin liposomal formulation was shown to be 26.9% bioaccessible [58]. Further, the same group revealed Fluronic-modified liposomes, and folated Fluronic-modified liposomes are nontoxic to the human KB cell lines [59].

Green tea polyphenols: Green tea comprises more than eight polyphenolic compounds (catechins) and is highly interesting among consumers for its potential health benefits, including antioxidant, antidiabetic, anti-inflammation properties. However, catechins are poorly absorbed in the body. When consuming green tea, only 5% of tea catechins reached the systemic circulation in rats and 1.68% in humans [60]. Liposomal formulation of catechin improves the GI stability and absorption in the body. Ezzat, et al. [61] developed chitosan-coated catechin liposomes and evaluated the pharmacokinetic properties in Wistar rats. Intestinal permeation from the in situ intestinal perfusion study revealed the rapid increase in intestinal absorption of chitosan-coated catechin liposomes (45.80 µg in 30 min); at the same time, conventional liposomal formulation and catechin solution absorbed only 18.26 and 7.36 µg, respectively. Similarly, the bioavailability of chitosan-coated catechin liposome was 1.37-folds higher than that of conventional liposomes and 2.12-folds higher than that of the catechin solution. Therefore, chitosan can directly interact with the tight junctions and facilitates the paracellular transportation and makes the chitosan-entrapped liposomes a promising approach for improving the intestinal absorption of bioactive compounds. Researchers worked with the different strategies to improve the bioavailability of catechins, including nanoencapsulation of catechins and nanoencapsulated catechin-based functional foods, and on different routes of delivering catechin. Recently, Fornasier, et al. [62] formulated catechin hexosomes (lipids aggregated in tubular arrangement) for the topical administration and found increasing penetration of catechins in the pig skin. To enhance the permeation properties, bile salts were used in the formulation and found that hexasomes with bile salts showed deeper transdermal penetration of the catechins than the hexasomes without bile salts.

Resveratrol: Resveratrol (trans-resveratrol; 3,5,4′-trihydroxystilbene) is a nonflavonoid polyphenolic compound found in grapes, red wine, and berries. Resveratrol is known for its health benefits, including anti-inflammatory, antiobesity, antioxidant, anticarcinogenic, and antiaging properties. On the downside, resveratrol is photosensitive and poorly soluble at the low GI phase and has low intestinal absorption and rapid metabolism. To overcome these limitations, nanosized formulations including liposomal entrapment of resveratrol can be employed. The liposomal formulation of resveratrol is gaining attention for its anticancer properties. A combination of anticancer bioactive compounds, resveratrol, and artemisinin in liposomes was evaluated for intestinal cancer cells [63]. The authors found that Eudragit-coated liposomes of both resveratrol and artemisinin reduced the viability of the HT-29 cells to 16% and 37% after 24 h exposure to 10 and 20 µg mL^−1^, respectively. The increased mortality of tumor cells was attributed to resveratrol’s multiple cellular targets affecting cellular proliferation and growth, including apoptosis, inflammation, invasion, angiogenesis, and metastasis [64].

Proliposomes: Proliposomes are a solidified form of liposomes formulated by removing water content in the liposomal suspension using different techniques: spray drying, freeze drying, vacuum drying, and fluidized bed drying. These dry granular proliposomes do not form a lipid bilayer structure during storing (increases stability), and they can be converted to liposomal formulation upon hydration. In a recent study, Jiao, et al. [65] optimized the supercritical fluid (ScCO_2_) technique for vitamin C (VC) proliposomes and reported that the optimal conditions were 25 MPa as pressure, 48 °C as temperature, and 0.25 as feeding ratio of vitamin C against phosphatidylcholine. This solid-state formulation ensures stability and is convenient for transportation, storage, and distribution. The solubility of proliposomes loaded with curcumin is estimated to be 98%, indicating the outstanding dispersible characteristics of proliposomes at the hydration step [66]. Further, proliposomes maintained an encapsulation efficiency of 92% after 30 days of storage at room temperature [67]. An in situ intestinal absorption study proved the increase in the absorption of liposomal formulations compared with the free form. Ren, et al. [68] reported a significant increase in absorption rate constant (2.3-fold) and absorptive fraction (1.4-fold) between the proliposome dispersion and its free form (quercetin was the model bioactive compound). Surprisingly, the in vivo pharmacokinetic study revealed a nearly 6.5-fold increase in C_max_ (maximum concentration) and a 12-fold increase in AUC (area under the curve) for the quercetin proliposomes. Such drastic increase in oral bioavailability was attributed to the solid formulation of quercetin proliposomes and their stability during GI transit [69]. Recently, Hızır-Kadı, et al. [70] improved the solubility of pollen phenolic extract (PPE) and improved the bioaccessibility by 2-fold compared with the conventional liposomes. However, the authors dissolved PPE in a few drops of ethanol during the proliposome production step, which may restrict the wide application of following this technique in an industrial scale.

Bile salt liposomes: To extend the application of liposomes further, bile salts are incorporated into the encapsulation process of liposomes, and such bile-salt-incorporated liposomes are called ‘nanobilosomes’. Bile salts in the ‘nanobilosomes’ further improve the aqueous solubility and dissolution rate and preserve the liposomes in the GI tract and enhance membrane permeability [71]. This ‘nanobilosome’ can be a promising technique to deliver the Biopharmaceutics Classification System (BCS) class IV bioactive compounds, which exhibit poor solubility and poor intestinal permeability. Mangiferin polyphenol is one of the poorly aqueous soluble (0.162 mg/mL solubility in water) and poorly intestinally permeable compounds (1.96 effective permeability in duodenum) [72]. Nanobilosome formulation improved the aqueous solubility by seven times and had better protection in the simulated gastrointestinal condition due to the electrostatic repulsive force between the bile salts in the formulation and the bile salts in the simulated intestinal fluid [73].

### 3.2. Niosomes

Niosomes are similar to liposome, both as a bilayer structure to entrap bioactive compounds. However, the bilayer for niosomes are made up of nonionic surfactants rather than phospholipids. Niosomes are used to encapsulate both hydrophilic bioactive (in the aqueous compartment) and lipophilic compounds (in the surfactant bilayer) [74]. The advantages of niosomes include biodegradability and compatibility with biological systems, and they possess tunable vesicle characteristics. Like liposomes, niosomes can entrap both hydrophilic (in vesicular aqueous core) and lipophilic (in the lipophilic domain of bilayers) bioactive compounds. Niosomes are low cost and have good stability and ease of storage of nonionic surfactants, which makes niosomes an effective alternative to liposomes. Further, surfactants forming niosomes are biodegradable, nonimmunogenic, biocompatible, and nontoxic.

#### 3.2.1. Physicochemical Stability and Encapsulation Efficiency

Niosomes are an effective formulation technique to maintain the stability of the encapsulated bioactive compound. Further, niosomes can entrap a higher quantity of bioactive compounds due to the equimolar ratio of cholesterol (nonionic surfactants), possible hydrogen bonding between the surfactants, and the entrapped drugs. Increasing cholesterol content in the niosomal preparation leads to the formation of rigid vesicles to entrap more bioactive compounds and provide sustained release characteristics [75]. Pando, et al. [76] measured the backscattering (BS) profile of niosomes upon storage for evaluating the stability of resveratrol-entrapped niosomes. The BS profile provides valuable information about the change in vesicle size distribution (due to destabilization mechanisms) and appearance of a creaming layer or clarification front with time. The authors observed a very slight variation of BS profiles for the niosomes prepared with Span 60: dodecanol (1:15) at 15,000 rpm. Zeta potential is strongly linked with the stability of niosomes. The magnitude and sign of the zeta potential determine the stability, and a magnitude of 30 mV (+ve or −ve) is a rule of thumb for a stable system [77]. Wagner, et al. [78] reported a zeta potential of niosomal formulation (encapsulated with vitamin D_3_ and ferrous sulfate) of −57.96 mV, which is far beyond the critical zeta potential threshold to maintain stability through mutual repulsion of particles. Niosomes were stable at 20 °C for a 20-day storage period. However, samples stored at 4 °C were observed with a reduction in particle size (1.4 to 0.9 µm) on the first day of storage due to thermal contraction. Additionally, a reduced thermal environment damaged the niosomal membrane and led to leakage of hydrophilic and hydrophobic compounds. Hence, there was a rapid reduction in the encapsulation efficiency for iron sulfate (25.1% to 23%) and vitamin D_3_ (95.1% to 86.90%). A niosomal preparation should always be stored above the precipitation temperature of the cargo concentration, or cargo solution concentration should be less than the precipitation limit for the desired storage temperature (cargo = bioactive in the hydrophilic region and bioactive in the hydrophobic region). Basiri, et al. [79,80] reported that α-tocopherol-loaded niosomes were highly stable in terms of particle size, zeta potential, and encapsulation efficiency during the storage. Even after 90 days of storage, there was only 30 nm increase in particles due to steric interactions among the large polar head group of surfactants (different combination of Span 60 and Tween 60), and insignificant variation in zeta potential (−30.1 to −28.8 mV only) resulted in lack of aggregation.

#### 3.2.2. GI Release and Bioavailability

When the ingested bioactive compound reaches the gastrointestinal tract, bioefficacy is a major concern due to gastrointestinal conditions such as digestive enzymes and bile salts. Tavano, et al. [81] suggested that niosomal formulation containing coencapsulation of nutrients (gallic acid/curcumin and ascorbic acid/quercetin) influences the physicochemical properties and entrapment efficiencies. Gallic acid and curcumin coencapsulated niosomes showed lower release profiles in simulated gastrointestinal conditions than the corresponding free compounds. When coencapsulating the bioactive compounds, bilayer composition and bioactive niosome interaction play an important role in the GI release profiles. Thus, after a 2 h exposure to intestinal pH, gallic acid is released from the vesicles at the same rate as curcumin, which confirms the complex interaction between them. Niosomal formulations have been reported for maintaining its stability in the GI tract. (−)-Epigallocatechin gallate (EGCG) is the most prominent flavonoid in green tea, but it loses it stability easily in the GI tract and is poorly absorbed in the intestinal epithelium cells. Liang, et al. [82] reported the gastrointestinal stability of EGCG niosomes through its z-average diameter, niosomes maintained their stability (60 nm) on 60 min exposure in a simulated gastric condition, and in the intestinal condition particle size was increased from 60 to 178 nm in 2 h. The drastic increase in particle size in the simulated intestinal condition is due to the addition of bile salts and pancreatin, which has a greater influence on niosomal stability. Further, a ferric reducing antioxidant power (FRAP) assay depicted that EGCG niosomes provided good protection throughout the intestinal tract.

## 4. Particulate-Based Systems

Particulate-based delivery systems have attracted huge attention among researchers and in the past two decades; it has transformed from scientific curiosity to active research interest. In the pharmaceutical sector, particulate-based drug delivery systems are most preferred as they are easy for oral administration, parenteral route either into the tissue or intravenously, and inhalation as a dry powder [83]. Particulate drug delivery systems are reported for increasing the bioavailability of therapeutic molecules that belong to classes II and IV of the Biopharmaceutics Classification System [84]. These compounds either are normally poorly water soluble or possess very low intestinal permeability.

### 4.1. Lipid Nanoparticles

In the middle of 1990s, a few research groups (Müller, Germany; Gasco, Italy; and Westesen, Germany) were working on alternative nanoparticles and reported solid lipids as an effective substitute to emulsions, liposomes, and polymeric nanoparticles. The main advantages of emulsion- and vesicular-based systems are the well-tolerated excipients, and they can be easily scaled up for bulk quantities. However, when compared with liposomes and emulsion formulations, solid particles possess more advantages, including protection of bioactive compounds against chemical degradation and flexibility in altering the release characteristics [85]. The advantages of solid particles and liquid formulations (emulsions and liposomes) were bridged for the birth of ‘lipid nanoparticles in solid state’.

Solid lipid nanoparticles (SLNs) are the most studied particulate-based delivery systems. SLNs are the matrix of lipid particles (solid) at both room and body temperature and can be formulated by replacing liquid lipid portions in an emulsion formula with solid lipids. Lucks and Muller [86] developed SLNs composed of 0.1% (*w*/*w*) to 30% (*w*/*w*) solid lipids dispersed in an aqueous medium and stabilized with a preferably 0.5% (*w*/*w*) to 5% (*w*/*w*) surfactant and achieved a mean particle size ranging from 40 to 1000 nm. Further, based on the incorporation of bioactive compounds, SLNs can be classified into three types [87]:

(i). Solid solution model: Bioactive compounds are molecularly dispersed in the lipid matrix. The cold homogenization technique is employed to prepare a solid solution model without using a solubilizing surfactant. There would be a strong interaction between the lipid and bioactive compounds. The SLNs of the solid solution model showed controlled release properties.
(ii). Bioactive-enriched shell model: The hot homogenization technique is employed to prepare SLNs. This model may not be suitable for the prolonged release of nutrients; however, it can be used to obtain a burst release of nutrients.
(iii). Bioactive-enriched core model: In this method, bioactives are solubilized in the lipid melt close to their saturation solubility. This model is suitable for the prolonged release of bioactive compounds [88].

Second-generation lipid particles (also called nanostructured lipid carriers (NLCs)) were prepared by blending solid lipids in liquid lipids (oils) preferably at a ratio of 70:30 up to a ratio of 99.9:0.1. The overall solid content of the second-generation lipid nanoparticles could be increased up to 95% and can be loaded with pharmaceutical and cosmetic bioactive compounds as well [89]. These lipid nanoparticles have multiple advantages over other nanodelivery systems, including lower chronic or acute toxicity due to physiological lipid, reduced usage of organic solvents in the preparation, protection of liable bioactive compounds, and the possibility of entrapping both hydrophilic and hydrophobic bioactive compounds and enhancing bioavailability [90].

### 4.2. Preparation of Solid Lipid Nanoparticles

SLNs are made up of solid lipids (triglycerides, fatty acids, and waxes), emulsifiers (poloxamer, polysorbate series, SDS), and water/solvent. Solid lipid nanoparticles can be produced using high-shear homogenization/ultrasonication, high-pressure homogenization, solvent emulsification–evaporation, solvent injection, microemulsion-based SLNs, supercritical fluid technique, spray drying method, and double emulsion method. Table 3 shows the main lipids and emulsifiers employed in the preparation of lipid nanoparticles.

**Figure 5:**
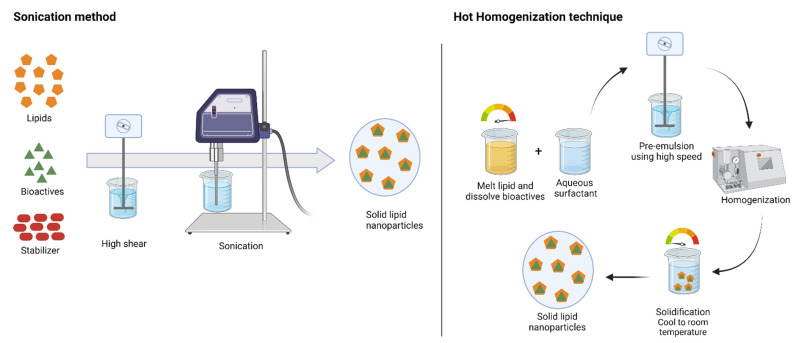
Schematic representation of popular solid lipid nanoparticle production method (created with Biorender.com).

High-shear homogenization/ultrasonication: High-shear homogenization and ultrasonication are the familiar tools for the preparation of SLNs in academic research due to their efficient and reproducible process. In this technique, the melted lipid in the aqueous phase with surfactants is homogenized using high-speed stirring to form emulsion and ultrasonicated to reduce the droplet size (see Figure 5). Gradual cooling of the prepared warm emulsion below the crystallization temperature forms the lipid nanoparticle dispersions [88]. Righeschi, et al. [91] used the high-shear homogenization process followed by ultrasonication for the preparation of curcumin SLNs. Pluronic F68-emulsified SLN formulation showed efficient encapsulation, particle size, zeta potential, stability, and prolonged release of curcumin in the intestinal environment. Increase in saturation solubility and release rate allows the curcumin SLN to reach high concentrations in the GI tract. Further, the Pluronic emulsifier has the ability to increase the permeability through the intestinal membrane and leads to the bioadhesion of curcumin in the GI wall.

High-pressure homogenization: High-pressure homogenization (HPH) is one of the most reliable and powerful techniques employed for emulsion preparation. In the mid-1990s, HPH was applied for the production of SLNs and NLCs and successfully achieved the stable formulations of lipid nanoparticles. HPH applies high pressure (100–2000 bar) on the feed liquid, which passes through a narrow gap. Due to very high shear stress and cavitation force, particles break down to the submicron range [92]. SLNs by HPH can be produced by two different approaches: hot homogenization and cold homogenization techniques (see Figure 5). For both approaches, the initial step starts with the dispersion/dissolution of bioactive compounds in the lipid melt. In the hot homogenization technique, the system temperature is maintained above the melting point of the lipid. The first step is the preparation of pre-emulsion for the lipid melt incorporated with bioactive compounds, and an aqueous phase containing a surfactant should be prepared using a high-shear device [92]. In the second step, homogenization of hot pre-emulsion uses high-pressure homogenization to achieve the desired particle size. The final step is cooling the emulsion to room temperature to generate SLNs/NLCs. Similarly, in the cold homogenization technique, bioactive compounds should be dispersed in molten lipid and rapidly solidified using dry ice or liquid nitrogen. Then the milled lipid matrix is dispersed in a cold aqueous surfactant mixture and homogenized to generate SLNs/NLCs [88].

Solvent emulsification–evaporation: Sjöström and Bergenståhl described the production of lipid nanoparticles by evaporating from the emulsion [107]. The preparation of SLNs involves two stages: (i) emulsification of lipophilic bioactive compounds (dispersed phase contains solvent) and (ii) evaporation of organic solvent and retention of the lipid nanoparticles by precipitation. Emulsification involves three stages: (i) dispersion of the lipophilic bioactive compound in an organic compound, a complex referred to as organic phase; (ii) pre-emulsion formation with the organic phase, surfactant phase, and aqueous phase; and (iii) nanoemulsification by passing the pre-emulsion through the high-pressure homogenizer to obtain nanodispersion. Upon evaporating the organic solvent, nanodispersion is formed by the precipitation of lipid in the aqueous phase and filtered to isolate the lipid nanoparticles [88,92].

Supercritical fluid (SCF) technique: A supercritical fluid is any substance where no distinct phase exists at a temperature and pressure above the critical point. Carbon dioxide has been most widely used due to its convenient critical temperature (T_C_), economical property, nonflammability, and nontoxicity [108]. In this technique, lipid nanoparticles were prepared by atomizing the emulsion in a supercritical fluid, and this technique is also called ‘supercritical fluid extraction of emulsions’. Similar to the solvent emulsification evaporation technique, the SCF technique is initiated by the preparation of nanoemulsion containing a lipophilic bioactive compound (dispersed phase: organic solvent) and atomizing the emulsion in SCF column [109].

### 4.3. Enhancing Oral Bioavailability of Curcumin through SLN/NLC Formulation

Curcumin is known for its wide range of health-beneficial properties; however, the poor bioavailability restricts the application of curcumin in commercial formulation. Yang, et al. [110] revealed the poor bioavailability of curcumin by orally administering the curcumin suspension to rats and evaluated the pharmacokinetic parameters, including maximum concentration (C_max_) of curcumin in the blood, time to reach C_max_ (T_max_), and area under the curve (AUC). Rats administered with curcumin suspension (500 mg/kg) orally achieved a C_max_ of only 0.06 ± 0.01 µg/mL, whereas rats after IV administration (10 mg/kg) achieved a C_max_ of 0.36 ± 0.05 µg/mL. The major reasons for the poor bioavailability includes poor solubility, physiochemical instability in the gut, metabolic instability in the liver, and poor intestinal permeability of curcumin.

The gastrointestinal tract is the favorite route of drug delivery, although many factors, such as harsh pH of the gastric environment, residence time, and solubility of the formulation, may affect the drug during this route. Numerous studies have reported the uptake of SLN and NLC formulation from the GI tract after oral administration and passage through the intestinal mucosa with improved bioavailability [111,112]. The mechanism behind the improved bioavailability of the SLN formulation is a two-stage process: first is the absorption of encapsulated bioactives into lacteals (lymphatic vessels of the small intestine) after oral administration; then the absorbed bioactive compounds are transported from lymphatic vessels to the thoracic lymph duct and eventually in the systemic circulation at the junction of the jugular and left subclavian vein. This transport mechanism avoids the first-pass hepatic metabolism and enhances bioavailability [112,113]. Sun, et al. [114] compared the pharmacokinetic parameters for the unformulated and SLN curcumin for intravenous administration in rats. Compared with free curcumin, SLN-formulated curcumin showed a significant difference in bioavailability, and the relative bioavailability of SLN to free curcumin was 125%. Although SLN formulation could enhance the oral bioavailability of curcumin, the burst release behavior of curcumin in the acidic environment has a negative impact on the bioavailability. To overcome this burst release, Ramalingam and Ko [115] developed chitosan-based curcumin SLNs. Chitosan is a cationic natural polysaccharide material showing high biocompatibility, biodegradability, and low toxicity. However, the application of chitosan for oral delivery is limited due to poor solubility above pH 5. Modifying the chitosan structure would be an ideal choice to improve solubility above acidic pH [116]. N-trimethyl chitosan (TMC) is a quaternized chitosan derivative reported for its excellent solubility over a wide pH range along with mucoadhesive and absorption-enhancing properties at a neutral pH [117]. In order to evaluate the absorption pattern of surface-modified chitosan-coated curcumin SLNs, the authors [115] orally administered the following curcumin formulations to mice: curcumin solution, curcumin-loaded SLNs (SLCNs), chitosan-coated SLNs (CH-SLNs), and N-trimethyl-chitosan-coated SLNs (TMC-SLCNs). SLCNs, CH-SLCNs, and TMC-SLCNs showed C_max_ values of 0.58 ± 0.03, 0.69 ± 0.16, and 1.21 ± 0.12 μg/mL, respectively. Surprisingly, the C_max_ of curcumin solution showed only 0.24 ± 0.05 μg/mL due to poor oral absorption, enzymatic degradation, and rapid elimination. Of the four formulations, surface-modified SLNs (TMC-SLCNs) showed higher relative bioavailability (23.07%) than the curcumin solution (1%). Increase in bioavailability for the TMC-SLCN formulation might be due to the coating material N-trimethyl chitosan, which has the ability to protect curcumin in the stomach environment and sustained release of curcumin in the intestinal tract. Similar to N-trimethyl chitosan, N-carboxymethyl chitosan is employed as a coating layer for SLNs (NCC-SLNs) and successfully used for encapsulating curcumin [118]. A pharmacokinetic study revealed a nearly 2.5-fold increase in C_max_ for the NCC-CLN formulation compared with the curcumin solution. From the T_max_ data, one can observe the prolonged release behavior of NCC-SLNs (2 h) compared with the C-SLNs (1 h) and curcumin solution (1 h). 

## 5. Self-Emulsifying Drug Delivery Systems

An emulsion-based delivery system is one of the promising technologies for encapsulating and delivering bioactive compounds [119,120]. However, it is extremely difficult to maintain stable emulsions, as the emulsion can lose its stability by one of the following mechanisms: gravitational separation, flocculation, coalescence, and Ostwald ripening [77]. Issues with the emulsion formulation can be coped with by spontaneously forming the emulsion system in the GI tract (also called self-emulsifying drug delivery system). Self-emulsifying drug delivery systems are a lipid-based technology with immense promise in enhancing oral bioavailability. SEDDS formulations contain isotropic mixtures of bioactive compounds, lipids, emulsifiers, and one or more hydrophilic cosolvents/co-emulsifiers. Once these ingredients reach the gut, they can form an emulsion system by the continuous agitation given by gastric wall motility [121]. Based on globule size in the dispersion, SEDDS can be further classified as self-micro-emulsified drug delivery system (SMEDDS) for the emulsion droplet size between 100 and 250 µm and self-nano-emulsified drug delivery system (SNEDDS) for the emulsion droplet size of less than 100 nm [122].

Mucoadhesive properties: The concept of mucoadhesion is gaining attention in the nutraceutical sector as it improves the bioavailability of administered bioactive compounds. Mucosa or the mucus membrane is a moist tissue covering the organ and cavities of the mouth, nose, eyelid, gut, and rectum [123]. The incorporation of mucoadhesive polymers in the SEDDS formulation is an ideal way to prolong the residence time of bioactive compounds. Mucoadhesive polymers such as chitosan and thiolated polymers can bind with mucosa by either noncovalent binding (adhesion with mucosa due to the polymer’s surface charge) or covalent binding (generating a covalent bond between the mucus layer and polymer). Incorporating mucoadhesive properties in the SEDDS will improve the bioavailability characteristics of the formulation. Therefore, any bioactive compounds need to permeate through the mucosal lining (in the GI tract) before being absorbed by the systemic circulation. With the permeation of macromolecules such as peptides, proteins are trapped by the mucus and degraded by digestive enzymes such as protease in the mucus layer [124]. Thus, administrating SEDDS with mucoadhesive polymers will spontaneously form droplets in the gut, and the formed droplets can penetrate the mucus layer effectively.

The incorporation of bioactives such as vitamin E and curcumin in SEDDS showed improved oral bioavailability due to their enhanced solubilization and cellular uptake [125,126]. Recently, Xu, et al. [127] identified the variation of the curcumin metabolites of curcumin in rats after administering three different curcumin formulations, namely, SEDDS, suspension, and a commercial phospholipid complex. The authors identified 34 curcumin metabolites using ultra-high-performance liquid chromatography/quadrupole time-of-flight mass spectrometry technique (UPLC–MS). The developed UPLC–MS procedure identified 9 curcumin metabolites from the in vivo samples. Surprisingly, only a small amount of SEDDS formulation is absorbed directly into the bloodstream, and most of the curcumin is transformed into metabolite curcumin glucuronide by intestinal UDP-glucuronosyltransferases (UGTs). UGTs are membrane-bound enzymes predominantly present in the liver, kidney, skin, brain, and intestinal epithelium. The main role of UGTs is to reduce the toxicity of administered compounds through glucuronidation and improve the biological activity [128]. Thus, a portion of curcumin was conjugated to glucuronide in the intestine and then reaches the portal vein and liver to form glucuronide/sulfate conjugates [129]. Although Xu, Tang, Zhang, Yang and Yuan [127] found an increase in bioavailability for rats administered with SEDDS formulation, the quantification of such curcumin metabolites for the different formulations is required to underpin the pharmacokinetic pathway of the SEDDS formulations.

## 6. Challenges and Future Perspective

A lipid-based delivery system is one of feasible formulation techniques to encapsulate and improve the oral bioavailability of food bioactive compounds. However, the production of lipid-based nutraceuticals without an organic solvent is still challenging. Further, research on supercritical fluid technology revealed high-quality lipid-based formulation without traces of an organic solvent. However, the application of a supercritical fluid technology for industrial production is also challenging.

Even though many published works reported on the functionality of lipid-based formulations, the global concern on the toxicological and regulation aspects needs to be answered for the wide commercialization of lipid-based products. Additionally, the acceptance of innovative lipid-based functional foods and nutraceuticals depends of the economic value, consumer perception, nutritional value, and market share of such products.

## 7. Conclusions

The increasing demand for healthy food products has pushed industries to develop nutraceutical products with high efficacy and bioavailability. Many nutraceutical compounds are poorly soluble in aqueous media, and this limits their wide application. A lipid-based nanocarrier is a brilliant idea to deliver those lipophilic compounds not only for improving stability but also for excellent oral bioavailability through different intestinal transport mechanisms. We should also consider that the market for ‘nanofoods’ is increasing, and research on characterizing new bioactive-loaded nanocarriers and the related toxicity risk is also increasing exponentially in recent years. The industrial production of nutraceutical products based on these research reports needs to be manufactured and transformed from lab to end users (consumers).

## Figures and Tables

**Table 1:** Summary of the lipid-based delivery system.

System	Definition	Advantages
Liposomes	Phospholipid bilayered vesicular systems having an aqueous core enclosed by one (unilamellar) or several (multilamellar) concentric phospholipid membranes.	Extensively studied lipid-based system. Employed for hydrophobic, hydrophilic, and amphiphilic bioactive compounds. Improved pharmacokinetics. Can be used for localized delivery. Commercialized for different nutraceutical formulations.
Niosomes	Similar to liposomes, but bilayers for the niosomes are made up of nonionic surfactants.	Biodegradability, compatibility with biological systems, and vesicle characteristics. Less expensive to formulate than the liposomes.
Solid lipid nanoparticles (SLNs)	SLNs are matrix lipid particles formulated by replacing liquid lipid portions in emulsion formula with solid lipids.	Reduced leakage of the incorporated compound. Protect the entrapped compound from harsh GI conditions. Application of organic solvents can be avoided. Large-scale production.
Nanostructured lipid carriers (NLCs)	Second generation of lipid nanoparticles containing a mixture of solid and liquid lipids.	Higher loading capacity. Affordable technique. Application of organic solvents can be avoided. Minimal leakage of bioactives during storage. Entrap both hydrophilic and hydrophobic bioactive compounds.
Self-emulsifying drug delivery system (SEDDS)	SEDDSs are isotropic mixtures of oil, surfactant, and cosurfactant that spontaneously form emulsion upon milk agitation.	Simple formulation technique. Thermodynamic stability. Spontaneous production of emulsion in the stomach motility. Enhanced absorption and increase in bioavailability.

**Table 2:** Clinical trials on lipid-based formulations.

Bioactives	Formulation	Title	Study Aim	Disease/Condition	Reference
Curcumin	Liposomes	Evaluation of liposomal curcumin in healthy volunteers	Safety and tolerability of increasing doses of intravenous liposomal curcumin	Health volunteers	NCT01403545 (ClinicalTrials.gov)
Curcumin	Liposomes	Comparison of curcumin bioavailability	Evaluate the bioavailability of curcumin of eight different formulations	Health volunteers	NCT03530436 (ClinicalTrials.gov)
Curcumin	Liposomes	A phase 1 study establishing the safety of intrapleural administration of liposomal curcumin (LipoCurc) as a palliative treatment for malignant pleural effusion	Investigate the safety of administering liposomal curcumin directly to the tumor site	Patient with long-term chest drain	ACTRN12620001216909 (ANZCTR.org.au)
Curcumin	LipiSperse^®^	A randomized double-blind placebo-controlled study to evaluate the effect of curcumin on BDNF levels in otherwise healthy adults	Investigate the bioavailable fraction of curcumin on BDNF levels in healthy adults	Brain-derived neurotrophic factor in healthy adults	ACTRN12621000104853 (ANZCTR.org.au)
Coenzyme Q10	Liposomes	A comparison of the plasma levels and safety of coenzyme Q10 from four different formulations in healthy adult volunteers	Evaluate the bioavailability of coenzyme Q10 in liposomal formulation	Patients with mild to moderate cardiovascular disease	ACTRN12616001527459 (ANZCTR.org.au)
Coenzyme Q10	SEDDS	The impact of micelle size and increased absorption of ubiquinone using a novel delivery system (AquaCelle^®^)	Evaluate the bioavailability of Coenzyme Q10 in SEDDS formulation	Healthy volunteers	[40]
EPA, DHA	SEDDS	A self-emulsifying omega-3 ethyl ester formulation (AquaCelle) significantly improves eicosapentaenoic and docosahexaenoic acid bioavailability in healthy adults	Evaluate the bioavailability of omega-3 fatty acid concentrations of SEDDS formulation	Healthy volunteers under the low-diet condition	[41]
Trans-resveratrol	LipiSperse^®^	Trans-resveratrol oral bioavailability in humans using LipiSperse™ dispersion technology	Pharmacokinetics of resveratrol–LipiSperse^®^ delivery complex	Healthy volunteers	[42]

SEDDS—self-emulsifying drug delivery system.

**Table 3:** Lipids and emulsifiers used for lipid nanoparticle production.

Bioactive Compounds	Lipids	Emulsifier	System	Model	Production Technique	Research Findings	Reference
Curcumin	Phosphatidylcholine, cholesterol	--	Liposome	In vitro release	Solvent dispersion and electrospray process	Prolonged and sustained release of curcumin was observed for 4 days with a low percentage (~37%) of curcumin release.	[93]
Curcumin	Bovine milk phospholipids, krill phospholipids	--	Liposome	In vitro digestion	Thin-film evaporation and ultrasonic dispersion method	Liposomes prepared by krill phosphate were easily digestible and showed higher bioavailability than the bovine milk phosphate liposomes.	[49]
Curcumin	Stearic acid and capric triglycerides	Tween 80 and Pluronic F127	NLC	In vitro digestion	Microemulsion + sonication	Nearly 41% of curcumin release from NLCs in simulated gastric medium up to 2 h and the drug release mechanism might be diffusion of curcumin from the matrix.	[94]
Curcumin	Tristearin	PEG10SE, PEG100SE	SLN	In vitro digestion	High-shear homogenization and ultrasound	PEG100SE-stabilized SLNs were highly permeable across the intestinal epithelium and improved the oral bioavailability by 6-fold compared with PEG10SE-stabilized SLNs.	[95]
Curcuminoids	Precirol^®^ ATO5 and Compritol^®^ 888 ATO	Poloxamer 188	NLC, SLN	In vivo pharmacokinetic study in mice	High-shear homogenization and ultrasound	Pharmacokinetic studies in mice revealed 4.48- and 3.41-fold increase in C_Max_ for SLN and NLC formulation, respectively.	[96]
Curcumin	Cetyl palmitate	Tween 60	NLC, SLN	Cell line studies in hCMEC/D3	High-shear homogenization and ultrasound	Transferrin-attached lipid nanoparticles can enhance the permeability across the blood–brain barrier (BBB). Transferrin is a transporter present in the luminal side of the brain and leads the receptor-mediated transcytosis across the BBB.	[97]
Curcumin	Compritol^®^ 888 ATO and oleic acid	Poloxamer 188, Tween 80, Span 80	NLC	In vivo antidepressant study in rats	Hot homogenization	Curcumin NLCs can be a neuroprotective agent. An in vivo study in rats improved the behavioral despair and enhanced the antidepressant and anxiolytic activity.	[98]
Curcumin	Triglycerides	Lecithin, Kolliphor HS15	NLC, SLN	In vitro release kinetics	Hot homogenization	The healing capacity of wounds by curcumin lipid formulations was assessed through scratch assay. After 24 h of exposure, the healing effects for NLCs and SLNs are 10.61% and 4.06%, respectively.	[99]
Curcumin	Beeswax	Lecithin, Tween 80	NLC, SLN	In vitro digestion	Hot homogenization	No cytotoxic effects were recorded for the undigested nanostructures and SLN formulation. However, a decrease in cell viability of NLC was attributed to the MCT oil digestion products for the cytotoxicity effects.	[100]
Quercetin	Phosphatidylcholine		Liposomes	Cell line study	Dispersion in aqueous phase followed by sonication	Eudragit-coated liposomes were safe in the intestinal cells without cytotoxicity.	[101]
Quercetin and linseed oil	Phosphatidylcholine		Liposomes	In vitro digestion	Ethanol injection method	Liposomal formulations were showing poor stability (flocculated) in the simulated gastrointestinal digestion. However, hydrogel beads of liposomes were stable in the digestion environment.	[102]
Resveratrol	Tripeptide lipid CDO	Sucrose laurate	Liposomes	In vitro release and in vivo antitumor activity	Thin-film hydration method	In vivo study on mice bearing breast cancer showed 5 mg kg^−1^ of resveratrol was more effective and 10 mg kg^−1^ completely inhibited the tumor growth.	[103]
Vitamin C, *β*-carotene	Cholesterol	Yolk lecithin	Liposomes	In vitro digestion	Ethanol injection method	During digestion, first, 10 min *β*-carotene was showing burst release, and then for the next 110 min, over 70% of bioactive substances were slowly released in the intestinal phase.	[104]
Rutin	Glycerol monostearate	Polyglycerol polyricinoleate, Span, and Tween	SEDDS double emulsion	In vivo pharmacokinetic study in rats	Mechanical stirring followed by dropwise addition of surfactant	The administered rutin SEDDSs spontaneously form O/O/W double emulsion in the GI tract and improve the solubility and absorption process. A nearly 1.76-fold increase in bioavailability was observed for the SEDDS formulation compared with the rutin suspensions.	[105]
Ferulic acid		Labrasol	SEDDS Microemulsion	In vivo pharmacokinetic study in rats	Manual mixing of formulation ingredients	SEDDS formulation improved the oral bioavailability of ferulic acid by 1.74-fold and strengthened the hypnotic efficacy. Brain tissues from the hippocampus and hypothalamus showed an increase in levels of 5-HT and 5-HIAA, which regulate sleep.	[106]

## Data Availability

Not applicable.
